# Effectiveness and biochemical impact of ozone gas and silica nanoparticles on *Culex pipiens* (Diptera: Culicidae)

**DOI:** 10.1038/s41598-024-67068-9

**Published:** 2024-08-19

**Authors:** Hend H. A. Salem, Shaimaa H. Mohammed, Randa I. Eltaly, Enayat M. Elqady, Eman El-said, Khaled H. Metwaly

**Affiliations:** 1 Zoology and Entomology Department, Faculty of Science, Al-Azhar University (Girls Branch), Cairo, Egypt; 2 Center of Plasma Technology, Al-Azhar University, Cairo, 11884 Egypt

**Keywords:** *Culex pipiens*, Ozone, Silica nanoparticles, Biochemical analysis, Scanning electron micrographs of the cuticle, Biochemistry, Physiology, Environmental sciences, Medical research, Nanoscience and technology

## Abstract

*Culex pipiens* (Diptera: Culicidae) is a vector of many serious human diseases, and its control by the heavy use of chemical insecticides has led to the evolution of insecticide resistance and high environmental risks. Many safe alternatives, such as ozone gas (O_3_) and silica nanoparticles (silica NPs) can reduce these risks. Therefore, O_3_ and silica NPs were applied to 3^rd^ larval instars of *Cx. pipiens* at different concentrations (100, 200, and 400 ppm) for different exposure times (1, 2, 3, and 5 min for O_3_ and 24, 48, and 72 h for silica NPs). The activity of some vital antioxidant enzymes as well as scanning electron microscopy of the body surface were also investigated. A positive correlation was observed between larval mortality % and the tested concentrations of O_3_ and silica NPs. O_3_ was more effective than silica NPs, it resulted in 92% mortality at 400 ppm for a short exposure time (5 min). O_3_-exposed larvae exhibited a significant increase in glutathione peroxidase, glutathione S-transferase, and catalase activities as well as the total antioxidant capacity. Scanning electron microscopy showing disruptive effects on the body surface morphology of ozone and silica NPs treated larvae. These results provide evidence that O_3_ and silica NPs have the potential for use as alternative vector control tools against *Cx. pipiens*.

## Introduction

Mosquitoes are considered one of the most economic and medical vectors of many diseases^1,2^. They are prevalent and capable of transmitting many diseases, causing millions of deaths every year^1,3^. In 2019, 229 million individuals were infected with malaria and approximately 409.000 individuals were killed worldwide^4^.

The extensive use of chemical insecticides has led to the development of insecticide resistance and high environmental risk; there is considerable defiance for using affordable alternatives to minimize these risks to the environment, non-targeted organisms, and humans^5^.

Ozone (O_3_) is generally recognized as a safe agent by the United States Environmental Protection Agency (USEPA) due to its unique properties as a high oxidizing agent used in the purification of drinking water, deodorizing agent, disinfecting capacity, and reduction of aflatoxin contamination^6,7^. O_3_ was successfully considered an antiseptic for agricultural products as well as to control microorganisms and insects, in addition to the elimination of insecticides, and organic and inorganic compounds^8^. O_3_ decomposed in water directly by reactions with unsaturated aromatic and aliphatic compounds, or indirectly by the production of intermediates called reactive oxygen species (ROS) such as hydrogen peroxide (H_2_O_2_), superoxide anion (O_2_^−^), and hydroperoxyl radical (HO_2_)^9^.

On the other hand, nanoparticles are considered new green biocidal insecticides for insect control because of their ability to prepare highly effective nanomaterials of diverse shapes and sizes^10^. For instance, silica NPs have been reported to have numerous applications in medicine and as pesticides in agriculture, but information on their effect on mosquitoes is limited. NPs mode of action depends on the desiccation of the insect cuticle through the physisorption of lipids, which causes cell lysis by damaging the cell membrane and ultimately insect death^11^.

The advantages of nanoparticles^12^ and ozone gas^13^ applications are that they are more environmentally friendly than conventional insecticides. Additionally, there are no residues of toxic chemicals and no danger of chemical mixing hazards. They also enhance ROS development in invading tissue^14–16^. ROS are essential players in insect immunity, and their overexpression can cause cellular damage followed by insect death^17,18^. Most cells have acquired many protective mechanisms to overcome the deleterious effects of excessive ROS inside the cell. Among these mechanisms are the production of antioxidant enzymes such as glutathione peroxidase (GPx), glutathione S-transferase (GST), and catalase (CAT)^18,19^.

Currently, there is limited research discussing the toxicity of O_3_ and silica NPs on mosquitoes, Therefore, the present study aimed to investigate the efficiency of ozone gas and silica nanoparticles on 3rd larval instars of *Cx. pipiens*.

## Materials and methods

### Insect collection

*Cx. pipiens* larvae were collected from four breeding sites located in Aswan, Egypt (the southernmost Egyptian Governorate bordering the Sudan Governorate) (24°28′49.5′′ N, 32°52′57.4′′ E).

A mosquito larvae dipper (250 mL) (Bio Quip products 2321 Glad wick Street Rancho Dominguez, CA 90220USA) was used to extract the larvae according to the method described by the WHO^3^. Three batches of mosquito samples were collected from the breeding sites within 4–5 days of each other. The collected larvae were placed in labeled stoppered plastic bottles leaving space about 1–2 cm for O_2_ supply, and the stoppers were removed every 120 min to provide fresh air to the specimens until reached the lab^3^. The collected larvae were examined and identified using taxonomic keys^20,21^.

The collected late 3rd and 4th instars of *Cx. pipiens* were transported to plastic containers containing dechlorinated tap water supplemented with fish food pellets and maintained under laboratory conditions [20 ± 2 °C and a photoperiod of 12:12 (L:D) h. Emerging pupae were collected from the larval trays each day and transferred into cups with dechlorinated tap water only and transported to cages for the emerging adults. The emergence of adult mosquitoes was maintained through three generations on 10% glucose and blood. 4th generation, 3rd larval instars were used for further experiments^22^.

### Experimental setup

#### Ozone gas production

**Figure 1:**
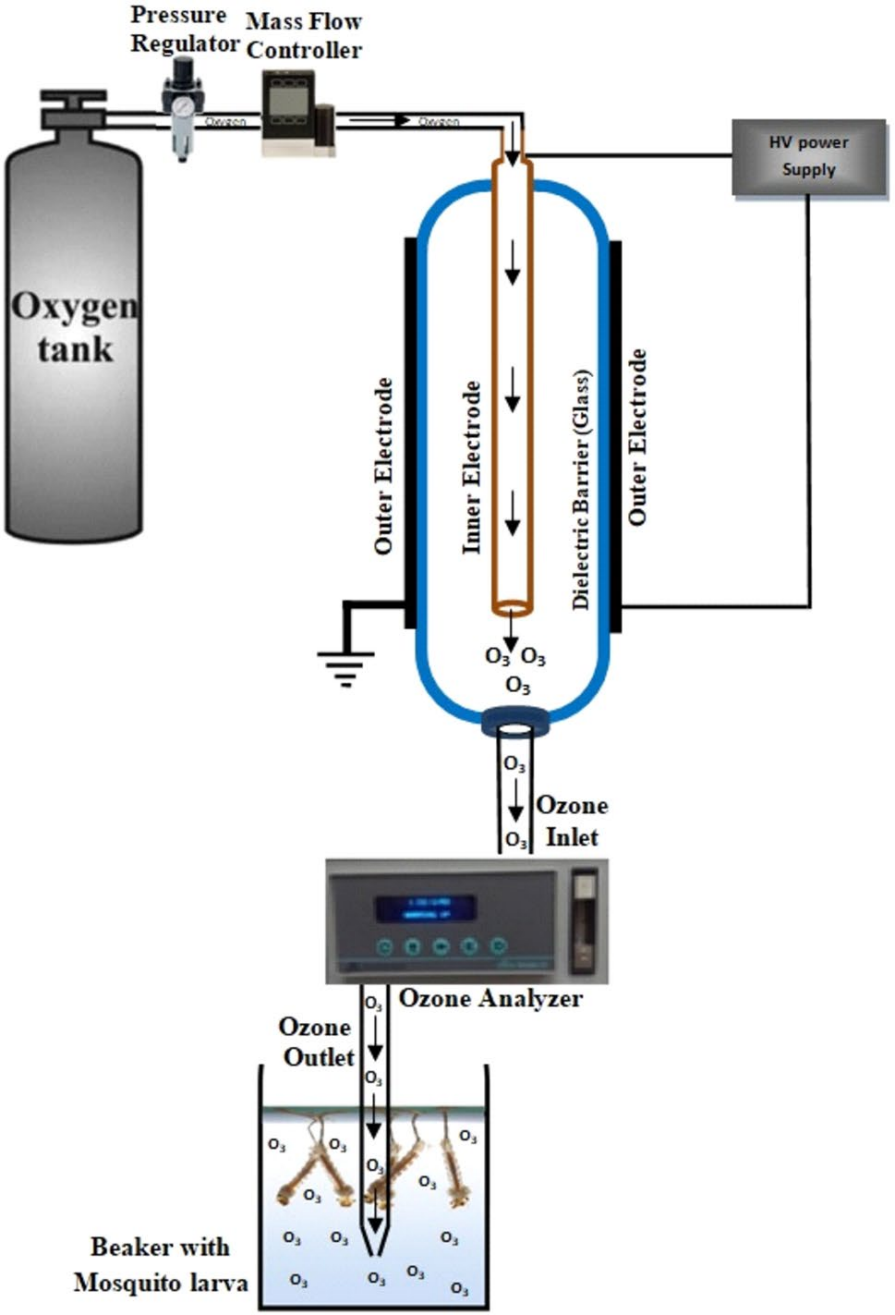
Schematic representation of the DBD ozone generation system. The generated ozone gas was pumped into a container with distilled water and containing mosquito larvae.

Ozone was generated from oxygen using a refined extra dry oxygen feed gas. A belt pan in the monitor controller enables the adjustment of different concentrations in a chosen range by an on-board plug-in sensor. Oxygen was pumped through a dielectric barrier discharge (DBD) reactor, which produced ozone as a byproduct. This kind of discharge is created by placing a discharge voltage between two coaxial electrodes that are separated by a glass dielectric and an open gap through which oxygen can flow. A filament discharge is created in this empty region, and the electrons produced there have enough energy to disintegrate the oxygen molecules that make up ozone. An ozone analyzer (Model H1-AFX-Instrumentation, USA) was used to measure the amount of ozone present inside the containers. The variable high voltage transformer used in the AC test set, or its input voltage, was 220 V at 50 Hz. There was a gap between the two outer and inner electrodes, which were connected to a voltage transformer as shown in (Fig. 1). A transformer control box (Variac Variable AC Power Transformer Regulator) was utilized to regulate the voltages needed to create ozone.

##### Characterization of electrical properties

**Figure 2:**
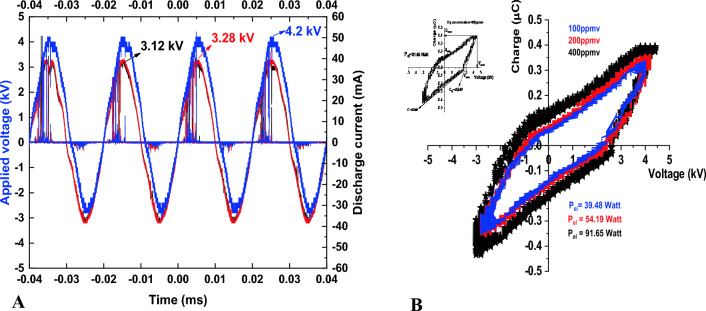
(**A**) Waveforms of the applied voltage to the reactor and ozone associated current measured at oxygen flow rate 0.1 (l/min); (**B**) Lissajous diagrams measured for ozone at a gas flow rate of 0.1 (l/min).

Data in (Fig. 2) illustrate the current–voltage oscillogram of the coaxial DBD reactor system (recorded at atmospheric pressure and room temperature). The filamentary mode discharges current in DBD when the applied voltage exceeds the breakdown voltage. In (Fig. 2A), the waveforms of the applied voltage to the coaxial DBD reactor and the associated current measured for ozone at an oxygen flow rate 0.1 (l/min) at different ozone concentrations of 100, 200, and 400 ppm are 3.12, 3.28, and 4.2 kV, respectively.

##### Power measurement method

The analysis of the power was performed according to the original work of Manley (1943), who utilized the voltage-charge Lissajous figures to characterize average consumed power through discharge^23–25^. The charge–voltage characteristic plot is illustrated in (Fig. 2). The capacitance of the effective discharge value was indicated by obtaining two distinct slopes of the Q–V plot. The reactor (coaxial dielectric barrier discharge) power formula is shown in equation^24^, where the total power (P_el_) is regarded as the operating frequency (ƒ) and the peak voltage V_max_. The Lissajous figure shows the minimum external voltage V_min_ at which ignition occurs, and the electric energy consumed per voltage cycle (E_el_) and the electric power (P_el_) can be estimated by the following relations^26^.

$$\begin{aligned} E_{{el}} = & 2(V_{{\max }} Q_{0} - Q_{{\max }} V_{0} ) \equiv {\text{Area }}\;{\text{of}}\;{\text{ (Q}} - {\text{V)}}\;{\text{ diagram}} \\ P_{{el}} = & \frac{{E_{{el}} }}{T} = fE_{{el}} \\ \end{aligned}$$

This result revealed that, the consumed power was 39.48 W at an applied voltage of 3.12 kV, 54.19 W at 3.28 kV, and 4.2 kV at 91.65 W. O_3_ concentration treatments were applied when ƒ was 50 Hz, as shown in (Table 1).

**Table 1:** Values of applied voltage and related power values at different ozone concentrations.

Ozone concentration (ppm)	Applied voltage (kV)	Electric power P_el_ (Watts)
100	3.12	39.48
200	3.28	54.19
400	4.2	91.65

#### Silica nanoparticles preparation

Hydrophilic silica NPs were purchased from Naqaa Company, Cairo, Egypt, and were prepared as described by El-Didamony et al.^27^.were.

##### Characterization of silica NPs and dynamic light scattering (DLS)

The size of a sample of silica NPs was analyzed by DLS Zeta Sizer (ZS), Malvern, United Kingdom.

**Figure 3:**
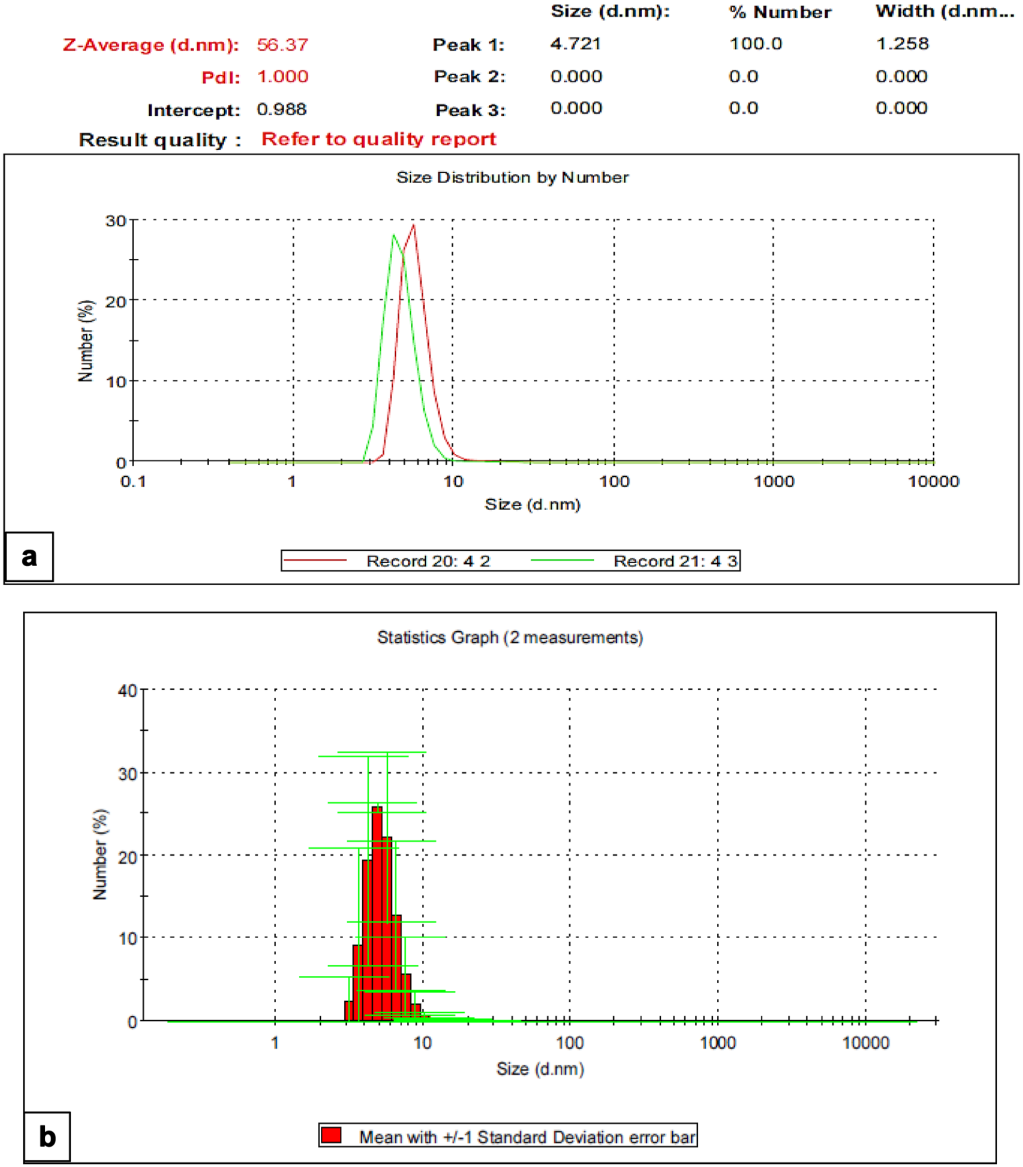
(**a**, **b**) Dynamic light scattering (DLS) of silica NPs.

DLS analysis revealed that the particle size of the silica NPs ranged from 3.122- 21.04 and the density size was 4.849 nm (25.8%). The PDI indicated nanoparticles with a uniform size distribution with a good polydispersity index (PDI) of 1.00 (Fig. 3a,b).

##### Transmission electron microscope (TEM) of the silica NPs

Nano silica was characterized by transmission electron microscopy (TEM) analysis, as outlined by^28^ and was prepared by placing a drop on carbon-coated copper grids and allowing water to evaporate. The size of the nanoparticles was determined from the TEM micrographs of JEOL JEM-1010 transmission electron microscope at 70 kV. The software (Advanced Microscopy Techniques, Danvers, MA) for the digital TEM camera was calibrated for size measurement of the nanoparticles. This analysis was carried out at the Regional Center for Mycology and Biotechnology (RCMB), Al-Azhar University.

**Figure 4:**
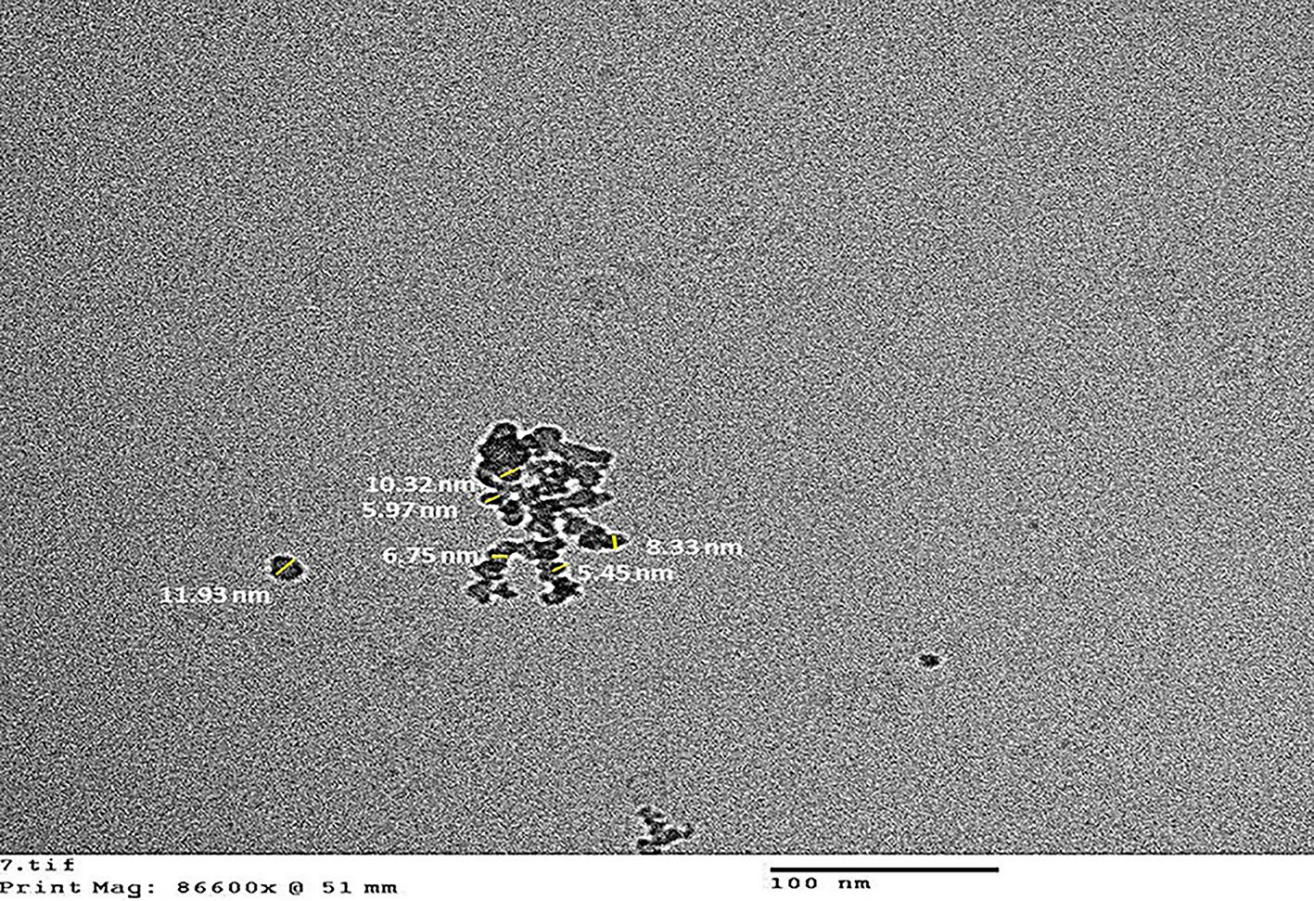
Transmission electron microscopy (TEM) image of the silica NPs.

TEM is a powerful method for detecting the morphology and size of nanostructures. TEM showed nearly spherical nanoparticles with nanometric range and a good size ranging from 5.14 to 12.2 nm. (Average 8.39 ± 2.35) (Fig. 4).

### Bioassay

#### Insects and ozonation

Ozonation was carried out in a specially modified plastic container (20 × 15) cm with two openings, one for gas entering and the second for gas exiting (Fig. 1). Each container contained 25 larvae in 250 ml of distilled water. Three ozone concentrations (100, 200, and 400 ppm) with different exposure times (1, 2, 3, and 5 min) were examined in this experiment. Four repetitions were examined for each condition. Each container was supplemented with fish food pellets (Tetra-Min, Germany) and maintained under optimum conditions of 20 ± 2 °C and a photoperiod of 12:12 (L:D) h. Similar conditions were prepared for the control groups but without ozonation**.** Mortality for all repetitions of all variants was recorded after 24 h.

#### Insects and silica NPs

The bioassay procedure was prepared as described by the WHO^28^. Three concentrations of silica NPs (100, 200, and 400 ppm) were adjusted in containers (20** × **15cm) with 250 ml water. Four replicates each with 25 larvae of *Cx. pipiens* (3rd larval instars) were used for each concentration. Fish food pellets (Tetra-Min, Germany) were supplied as larval food during experimentation. under the optimum conditions 20 ± 2 °C and a photoperiod of 12:12 (L:D) h^29^. Similar conditions were prepared for the control groups with distilled water only. Mortality was checked at 24, 48, and 72h post-exposure. The other four replicates were tested without any silica NPs as the control experiment.

### Biochemical experiment

The activities of GPx, GST, CAT, and T-AOC in *Cx. pipiens* 3rd instar larvae were investigated at 24h following exposure to 100, 200 and 400 ppm of O_3_ gas or silica NPs. Four replicates per treatment, each with 25 larvae were set up for each enzyme. The absorbance of the colored substance was measured by a double-beam ultraviolet–visible spectrophotometer (Spectronic 1201, Milton Roy Co., USA). Larval tissue homogenate was prepared in 40 mL of ice-cold phosphate buffer (0.05 M, pH 7.0) by a homogenizer at 30 W, for 10 s. The homogenate was centrifuged for 30 min (4 °C, 10,000 × g), and the resulting supernatant was used as an enzyme solution. The protein content of the enzyme solution was determined according to the method described by Bradford (1976)^30^. All chemicals used were obtained from Sigma.

#### Glutathione peroxidase (GPx)

GPx activity was determined according to the procedure given by Feston, (2015)^31^. To a spectrophotometer sample cuvette, 10 μl of sample and 50 μl of a supplied mixture of NADPH, glutathione, and glutathione reductase were added. The reading was adjusted to zero at 340 nm. To initiate the reaction, 20 μl of cumene hydroperoxide were added to the sample cuvette then absorbance was read at 340 nm for 5 min at 24°C. The enzyme activity was expressed as a change in absorbance/min/g sample.

#### Glutathione S-transferase (GST)

Glutathione S-transferase (GST) catalyzes the conjugation of reduced glutathione (GSH) with 1-chloro 2,4-dinitrobenzene (CDNB) via the –SH group of glutathione. The conjugate, S-(2,4-dinitro-phenyl)-L-glutathione could be detected as described by Habig et al*.*(I974)^32^. The reaction mixture consisted of 1 ml of potassium salt in phosphate buffer (pH 6.5), 100 µl of GSH and 200 µl of larval homogenate. The reaction started with the addition of 25 µl of the substrate CDNB solution. The concentration of both GSH and CDNB were adjusted to be 5 and 1mM, respectively.

Enzyme and reagents were incubated at 30°C for 5 min. The increment in absorbance at 340 nm was recorded against a blank containing all the enzymes except the enzyme to determine the nanomole of substrate conjugated/min/larva using a molar extinction coefficient of 9.6/mM/cm.

#### Catalase (CAT)

CAT activity was measured using a bio diagnostic kit No. CA 2517 as described by Aebi (1984)^33^. The reaction started with a known quantity of hydrogen peroxide, and then stopped after 1 min with catalase inhibitor. The presence of peroxidase led to the reaction of H_2_O_2_ with 3,5-dichloro-2-hydoxybenzene sulfonic acid and 4-aminophenazone to form a chromophore with a color intensity inversely proportional to the amount of catalase in the sample. The absorbance was measured at 510 nm. The CAT activity was expressed as millimoles of H_2_O_2_ reduced/minute/milligram of protein.

#### Total antioxidant capacity (T-AOC)

T-AOC was measured by using a bio-diagnostic kit (No. TA 2513). The antioxidants in the examined sample were reacted with a known quantity of exogenous hydrogen peroxide by eliminating of a certain amount of H_2_O_2_. The residual hydrogen peroxide was determined calorimetrically at 505 nm by an enzymatic reaction that involves the conversion of 3,5, dichloro-2-benzenesulfonate to a colored product. T-AOC activity was expressed as mg ascorbic acid equivalent/gm sample.

### Scanning electron microscopy examination

Scanning electron microscopy (SEM) was used to investigate the effect of O_3_ and silica NPs particles on the cuticle of 3rd instar mosquito larvae after 24 h following O_3_ or silica NPs exposure. The larvae were washed with buffered phosphate (pH 7.2), then fixed in 2.5% glutaraldehyde, and dehydrated in a serial dilution of ethanol as described by Attia et al*.*^34^. The larvae were imaged using a scanning Electron Microscope (JSM 5200, Electron Probe Microanalyzer, JEOL, Japan).

### Statistical analysis

The mortality data were estimated by using probit analysis through SPSS V23 (IBM, USA). The results of the biochemical assays are presented as the mean ± SE. The data were statistically analyzed by ANOVA using SPSS V. 23. Statistical significance was analyzed using Tukey’s test at *p* ≤ 0.05 probability level. All graphs were made using Excel 2010 (v12.0).

### Ethical approval

The present study does not have any test/evaluation in humans performed by any of the authors in this article.

## Results

### Larval susceptibility to O_3_ gas and silica NPs

**Figure 5:**
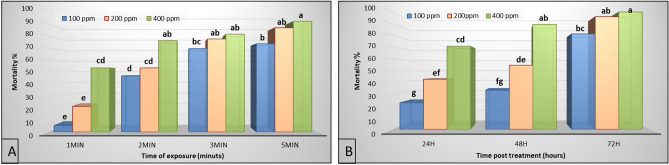
Percent mortality of *Cx. pipiens* larvae after O_3_ gas treatment (**A**). Percent mortality of *Cx. pipiens* larvae after silica NPs treatment (**B**). Significant differences are indicated by different letters (Tukey’s test at *P* ≤ 0.05).

The results showed that *Cx. pipiens* larvae exhibited high susceptibility to low concentrations of O_3_ gas with percent mortalities 46.67, 53.33, and 76.00% for 100, 200, and 400 ppm, respectively after 2 min post-exposure (PE). At 3 min PE, the percent mortalities reached 69.33, 77.33, and 81.33%, for 100, 200, and 400 ppm respectively. While at 5 min PE, the percent mortalities reached 73.33, 86.67, and 92.00% for 100, 200, and 400 ppm, respectively, after 5 min of O_3_ exposure (Fig. 5A).

Similarly, different concentrations of hydrophilic silica NPs (100, 200, and 400 ppm) exhibited a highly toxic effect on *Cx. pipiens* larvae. The percent mortalities at 24 h post-treatment (PT) were 22.67, 42.67, and 70.79%, respectively. At 48 h PT, the percent mortalities were 33.33, 54.66, and 89.33%, respectively. While the mortalities percentage at 72 h PT were 81.33, 96.00, and 100.00%, respectively (Fig. 5B).

### Biochemical assay

**Figure 6:**
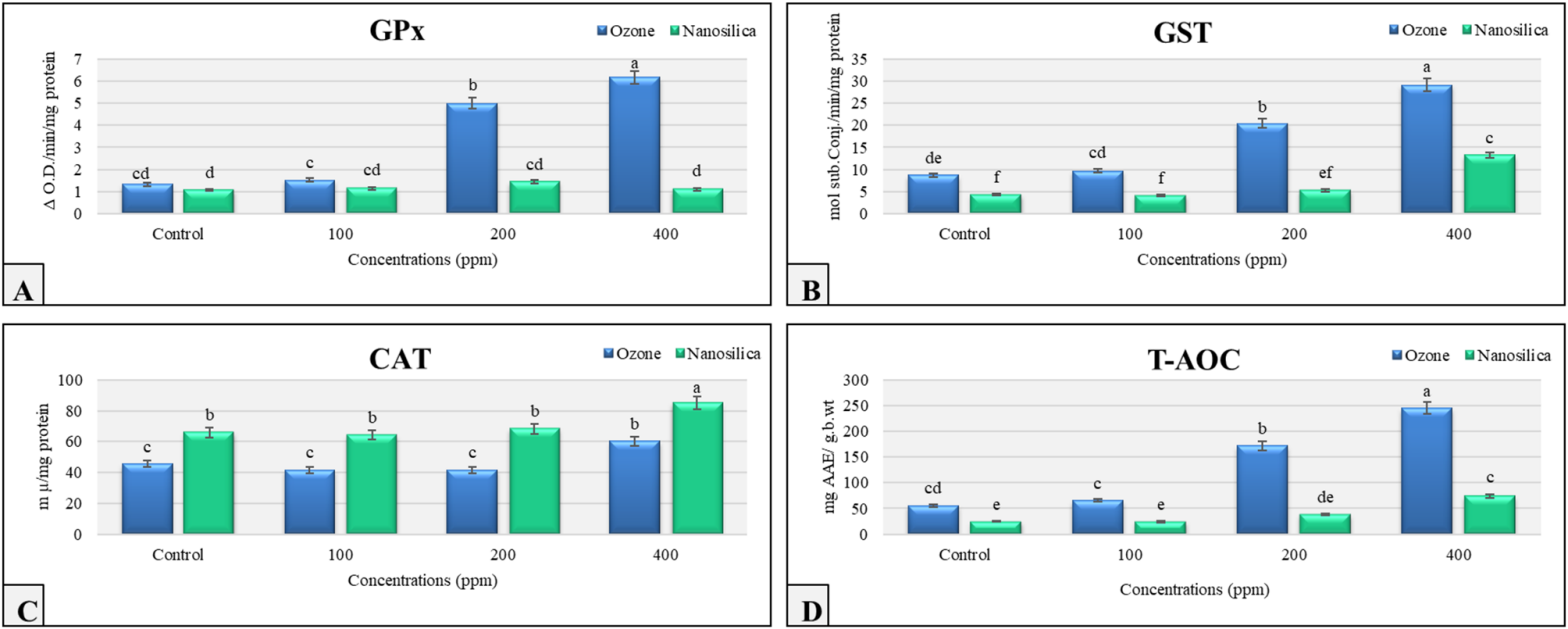
The activities (mean ± SE) of the antioxidant enzymes, glutathione peroxidase (**A**), glutathione S- transferase (**B**), catalase (**C**), and antioxidant capacity (**D**) in 3rd larval instars of *Cx. pipiens* after exposure to 100, 200, or 400 ppm O_3_ and silica NPs. Significant differences are indicated by different letters (Tukey’s test at *p* ≤ 0.05).

(Fig. 6) shows the activities of the antioxidant enzymes glutathione peroxidase, glutathione transferase, catalase, and the antioxidant capacity of the 3rd larval instar of *Cx. pipiens* after exposure to 100, 200, or 400 ppm of O_3_ or silica NPs.

#### Glutathione peroxidase (GPx)

Data in (Fig. 6A) shows a positive correlation between GPx activity and concentrations in ozone-treated larvae, while GPx activity in silica NPs-treated larvae wasn’t significantly different at any of the tested concentrations compared that in the control group (*p* < 0.05).

#### Glutathione S-transferase (GST)

The results in (Fig. 6B) shows a significant increase in GST activity by increasing O_3_ concentration while in the case of silica NPs-treated larvae, the only significant increase was observed at the highest concentration tested (400 ppm) compared to that of the control(*p* < 0.05).

#### Catalase (CAT)

Compared with that in the control larvae, the catalase activity in 3rd instar *Cx. pipiens* larvae in the ozone and silica NPs treatment groups didn’t significantly differ from that in the control group at 100 and 200 ppm but increased significantly at 400 ppm (*p* < 0.05) (Fig. 6C).

#### The antioxidant capacity

The antioxidant capacity of the ozone-treated larvae at all concentrations was significantly greater than that of the silica NPs-treated larvae (Fig. 6D), which was significantly greater only at 400 ppm than in the control larvae (*p* < 0.05).

### Scanning electron micrographs

**Figure 7:**
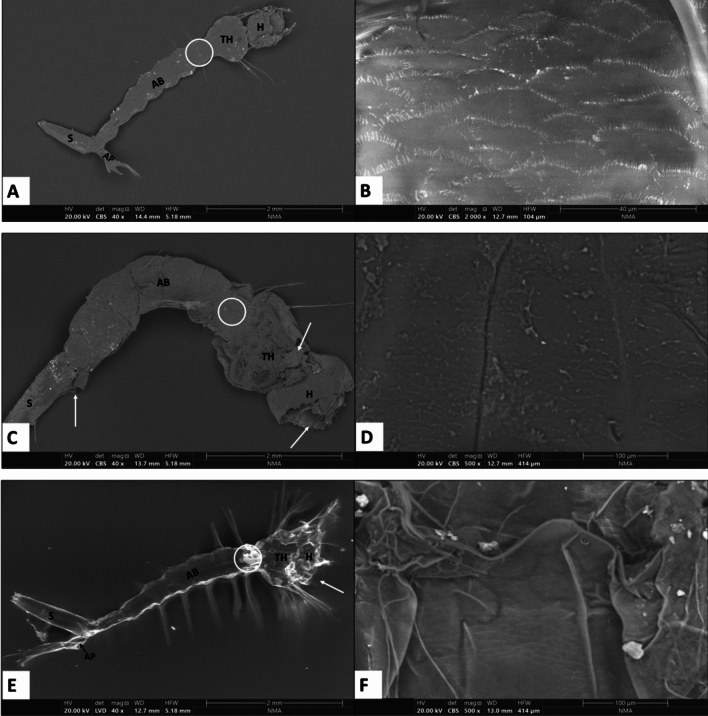
Scanning electron micrographs of 3rd instar larvae showing general body morphology of the head (H), thorax (TH), abdomen (AB), siphon (S) and anal papillae (AP). (**A**) and (**B**) show the control (untreated larvae). (**C**) and (**D**) show treated larvae.(**E**) and (**F**) show magnification silica NPs-treated larvae. (**B**, **D,** and **F** show magnifications of the circled area of the first abdominal segment). The damaged parts indicated by arrows.

Figure 7shows the ultrastructure of *Cx. pipiens* 3rd larval instar. The general body morphology of the normal larvae included the head, thorax, abdomen, siphon, and anal papillae (Fig. 7A) as well as the normal cuticle surface (Fig. 7B). While the ultrastructure of the larval cuticle exposed to O_3_ showed extensive damage represented by an explosion in the head region, loss of anal papillae, and swelling of the body because of water entry (Fig. 7C, as well as cracking of the surface of the body (Fig. 7D). However, silica NPs treated larvae show high shrinkage in the whole body (Fig. 7E,F).

## Discussion

Mosquitoes play a vital role in the transmission of many diseases such as malaria, filariasis, and Rift Valley fever^35^. The development of insecticide resistance has necessitated the use of alternative control strategies and tools. O_3_ and silica NPs are more eco-friendly agents than traditional insecticides^9^.

In the current study, there was a positive correlation between O_3_ and silica NPs concentrations and mortality %. O_3_ was more effective than silica NPs by inducing 92% mortality at 400 ppm for a short exposure time (5min). O_3_ is an unstable, water-soluble gas that rapidly decomposes to form oxygen and highly reactive free radicals^36^. Accordingly, O_3_ is a powerful oxidizing agent, used to disinfect water for short exposure times without any negative effects on associated aquatic organisms^36,37^. Many researchers have investigated the effectiveness of ozone on many insect species. The complete mortality of the biting gnats *Culicoides variipennis* ( Order: Diptera) was observed after an hour of ozone exposure at 600 ppm^38^. However, the mortality of adult and larval stages of *Galleria Mellonella* reached 100% after 7 h of exposure^39^. Similar results showed the toxic effect of ozone gas on many stored products insects^40,41^.

Similarly, hydrophilic silica NPs showed high efficacy against *Cx. pipiens* larvae. Silica NPs are among the most widely used natural materials as bioinsecticides^11^. Many authors have reported the effectiveness of silica NPs on *Cx. Pipiens*^42^ or on *Cx. Quinquefasciatus*^43^. Silica NPs are the most natural materials as bioinsecticides effectiveness of silica NPs on *Cx. pipiens* or on *Cx. quinquefasciatus.*

Ozone is a strong oxidative agent that targets the respiratory system of insects^44^ Ozone decomposes into the natural component of the atmosphere O_2_ and highly reactive oxygen atoms^45^. This single free oxygen atom reacts with the cell membranes of organisms and disrupts their normal cellular activity^46^. Once it enters cells, it oxidizes all the essential components and causes oxidative stress which damages the cell and leads to death^47^. When ozone decomposes in water, it forms the free radicals of hydrogen peroxyl (HO_2_) and hydroxyl (OH), which have great oxidizing capacity and form reactive oxygen species (ROS) inside the cell^7,48,49^.

On the other hand, silica is one of the most natural materials on earth. Silica nanoparticles have several applications, including biocidal activity against many pests^48,50,51^. Silica NPs mode of action involves desiccation of the insect cuticle by physisorption of lipids and these NPs are also expected to cause damage to the cell membrane resulting in cell lysis and ultimately death of the insects^10^. However, cells tend to eliminate excessive ROS through the production of antioxidant enzymes^10,17^. The antioxidant defense system in invertebrates could be more important than that in vertebrates. Severe oxidative stress is known to initiate a cascade of antioxidant responses that include GPx, CAT, GST and the antioxidant capacity in general^29,52^. In this study, a significant increase in GPx, GST, and CAT enzyme activities as well as T-AOC in ozone-exposed mosquito larvae was observed. In contrast, the only an increase of these enzymes activities was observed at high concentrations (400 ppm) of silica NPs exposed larvae. Ozone is commonly used in water treatment operations as well as odor and microorganismal remediation. The free HO_2_ and OH that are formed due to O_3_ decomposion play an active role in the disinfection process^12,46^. Increased production of hydrogen peroxide induced the activity of the antioxidant enzymes^53^. Antioxidant enzymes, such as glutathione peroxidase (GPx), catalase (CAT), and peroxiredoxin (Prx) are essential components in cells for elimination of excessive reactive oxygen species (ROS) in cells^17^. The effect of three different concentrations of commercial silver nanoparticles on the expression of antioxidant and detoxification genes in *C. riparius* for 24 h resulted in the upregulation of catalase, glutathione peroxidase, and glutathione S-transferase levels^54,55^.

The ultrastructure of the larval cuticle exposed to O_3_ showed extensive damage represented an explosion in the head region, cracking of the surface of the body, and loss of anal papillae as well as swelling of the body because of water entry. However, silica NPs- treated larvae exhibit high shrinkage in the whole body. Even if a wide number of ozone or nanoparticle research have been tested against mosquitoes, their impact on mosquito tissues or cuticle is still scarce.

Insect cuticle generally consists of three layers (exocuticle mesocuticle and endocuticle). The cuticle is a multifunctional coat that defines and stabilizes the shape of the body, appendages, and internal organs and prevents dehydration and infection^54^. The epicuticle layer of the exocuticle contains a wide range of saturated, unsaturated, and branched hydrocarbons broadly defined as cuticular hydrocarbons (CHCs)^56^. Ozonolysis of live *Drosophila suzukii* significantly reduces the total amount of unsaturated hydrocarbons that are responsible for wax formation^57^. This destruction in the wax layer affects the water permeability and flexibility of the epicuticle^54^. Moreover, ROS gases may have quite different chemical effects on chitin, ozone depolymerizes chitin and reduces its molecular weight^58^. The integument of *R. sanguineus* females exposed to 3 mg/L ozonized water for 5 min showed a decrease in the thickness of the cuticle and subcuticle layers with duplication of the epithelium layer after 24 h while after 48 h, the subcuticle layer was thinner and contained vacuolated epithelial cells^59^. This probably happened due to the ozonated water, which was able to stimulate the cells to secrete cuticular components faster and in greater quantity to reinforce the cuticular layers and minimize damages caused by the entry of toxic substances inside the organism^56^. Moreover, silica NPs absorbed into the cuticular lipids of the insects through physiosorption, causing desiccation and subsequently insect death^60^. In agreement with our findings, the shrinkage of *Aedes aegypti* cuticle after treatment with Ag nanoparticles was observed^61^.

## Conclusion

The current study proposed finding an effective and inexpensive method to reduce the spread of disease vectors. Ozone and silica nanoparticles are eco-friendly alternatives to chemical insecticides for humans and the environment. In this study, it has been observed that both ozone and silica nanoparticles were effective against *Culex pipiens*. Moreover, ozone was faster and more virulent in low concentrations than silica nanoparticles, in addition to its obvious deleterious effects on the cuticle and some changes in antioxidant enzymes of the insect in addition to its known passive role in the purification of polluted water, so we recommend expanding its application in mosquito breeding sites.

## Data Availability

The datasets collected and/or analyzed during the current study are available from the corresponding author on request. The corresponding author had full access to all the data in the study and took responsibility for the integrity of the data and the accuracy of the data analysis.
